# Permanent pacemaker dependency in patients with new left bundle branch block and new first degree atrioventricular block after transcatheter aortic valve implantation

**DOI:** 10.1038/s41598-021-03667-0

**Published:** 2021-12-21

**Authors:** Bonnie Hartrampf, David Jochheim, Julius Steffen, Thomas Czermak, Sebastian Sadoni, Erik Lemmermöhle, Ina Klier, Heidi L. Estner, Steffen Massberg, Julinda Mehilli, Korbinian Lackermair, Stephanie Fichtner

**Affiliations:** 1 Department of Medicine I, University Hospital Munich, Ludwig Maximilians University, Marchioninistr. 15, E81377 Munich, Germany; 2 DZHK (German Centre for Cardiovascular Research), Partner Site Munich Heart Alliance, Munich, Germany; 3 Department of Cardiac Surgery, University Hospital Munich, Ludwig Maximilians University, Munich, Germany; 4 Department of Cardiac Surgery, Klinikum Augustinum, Munich, Germany; 5 Medizinische Klinik I, Landshut-Achdorf Hospital, Landshut, Germany

**Keywords:** Cardiac device therapy, Interventional cardiology

## Abstract

Conduction disorders with need for permanent pacemaker (PPM) implantation remain frequent complications after transcatheter aortic valve implantation (TAVI). Up to 22% of PPM after TAVI are implanted for new onset left bundle branch block (LBBB) and atrioventricular block (AVB) I. However, clinical benefit and predictors of ventricular pacing in TAVI patients receiving PPM for this indication remain unclear. We retrospectively evaluated pacemaker interrogation data of patients who received a PPM post TAVI for new LBBB and new AVB I. The primary endpoint of this study was relevant ventricular pacing (ventricular pacing rate: Vp ≥ 1%) at the first outpatient pacemaker interrogation. Secondary endpoints were predictors for relevant ventricular pacing. At the first pacemaker interrogation (median follow up at 6.23 [2.8–14.8] months), median ventricular pacing frequency was 1.0% [0.1–17.8]. Out of 61 patients, 36 (59%) had Vp rates ≥ 1%. Patients with frequent ventricular pacing showed longer QRS duration (155 ms ± 17 ms vs. 144 ms ± 18 ms, p = 0.018) at the time of PPM implantation and were less likely treated with a balloon-expandable Edwards Sapiens Valve (39% vs. 12%, p = 0.040). Our findings suggest that the majority of patients with new LBBB and new AVB I after TAVI show relevant ventricular pacing rates at follow up. Further prospective studies are necessary to identify patients at higher risk of pacemaker dependency.

## Introduction

Over the past decade, transcatheter aortic valve implantation (TAVI) has been established as a safe and effective treatment for severe aortic stenosis, particularly in elderly and high- or intermediate risk patients^1^. Recent data also suggest TAVI to be an alternative with non-inferior or even superior short-term outcome in low risk patients compared to surgical valve replacement (SAVR)^2,3^. However, conduction disorders remain a frequent complication and often necessitate permanent pacemaker (PPM) implantation. This poses an additional risk for complications and is associated with longer hospital stay and higher costs^4^. After 6 months, the overall complication rate after implantation of a cardiac implantable electronic device is 9.5%^5^. In the long term, high ventricular pacing rates (Vp) can cause deterioration of left ventricular function and thus may limit the beneficial effects of TAVI^6,7^. This may limit the success of TAVI especially in younger, low risk patients. Considering potential risks and additional costs, it is of particular importance to identify which patients benefit from PPM implantation after TAVI.

Previous studies could show that patients with high degree or complete AVB often do not recover from post-procedural conduction disturbances and require frequent ventricular pacing. However, up to 22% of PPM after TAVI are implanted for preventive reasons in patients with new onset left bundle branch block (LBBB) and atrioventricular block (AVB) I^8–10^. Up to now, clinical benefit and pacemaker dependency in these patients remain controversial. While two earlier studies found no relevant ventricular pacing rates in patients with new LBBB after 3 months^11,12^, the largest study to date enrolling 23 patients reported that more than half of the patients with LBBB and AVB I require ventricular pacing at follow-up^13^.

The aim of this study was to determine atrial (Ap) and ventricular pacing rates (Vp) in a larger cohort of patients with new LBBB and new relevant AVB I after TAVI at the first outpatient pacemaker interrogation and to identify predisposing factors for frequent ventricular pacing.

## Methods

### Patient population and design

This study was conducted as a retrospective analysis of data from the Every TAVI registry (ClinicalTrials.gov Identifier: NCT02289339). It was approved by the Ethics Committee of Ludwigs- Maximilians- University. Due to the retrospective design of the analysis, the need for informed consent was waived by the Ethics Committee of Ludwigs-Maximilians-University. All patients who received PPM after TAVI between 2014 and 2018 were reviewed for inclusion. Patients were included for analysis, if PPM implantation was performed for new LBBB **and** new AVB I. According to hospital policy, patients had continuous ECG monitoring for up to seven days. Patients with LBBB and AVB I post TAVI received a pacemaker if: (a) QRS durations continued to increase within the monitoring period (b) QRS > 130 ms and PQ > 250 ms occurred during the first seven days after TAVI. Exclusion criteria were high- degree AVB or bradycardia due to atrial fibrillation. At the first outpatient follow up it was determined how many of the patients showed relevant ventricular pacing. Patients with and without relevant pacing were compared for baseline and procedural characteristics. All methods were performed in accordance with the relevant guidelines and regulations.

### End points

The primary endpoint was defined as relevant ventricular pacing at the first ambulatory pacemaker interrogation. According to previous publications^11^, relevant ventricular pacing was considered as Vp of 1% or above.

Ap and Vp were evaluated. Patients without (group 1 = Vp < 1%) and with relevant ventricular pacing (group 2 = Vp ≥ 1%) were compared for baseline and procedural characteristics and pacemaker settings to detect predictors for need of ventricular pacing in this cohort. Pre- and post- procedural ECG were compared for conduction disorders and arrhythmia.

### Statistics

Statistical testing was performed using SPSS (IBM® SPSS Statistics Version 25.0). Data for continuous variables were expressed as median and interquartile range or in mean ± standard deviation, if appropriate. Comparison was calculated by Mann–Whitney-U or t-test. Categorical Data were compared using the Chi-Square test or, if appropriate, Fisher’s Exact test. A p-value of < 0.05 was considered statistically significant.

### Ethics approval

Ludwig-Maximilians- University, Ethics Committee, Project Nr. 17662.

## Results

### Patient characteristics

**Figure 1:**
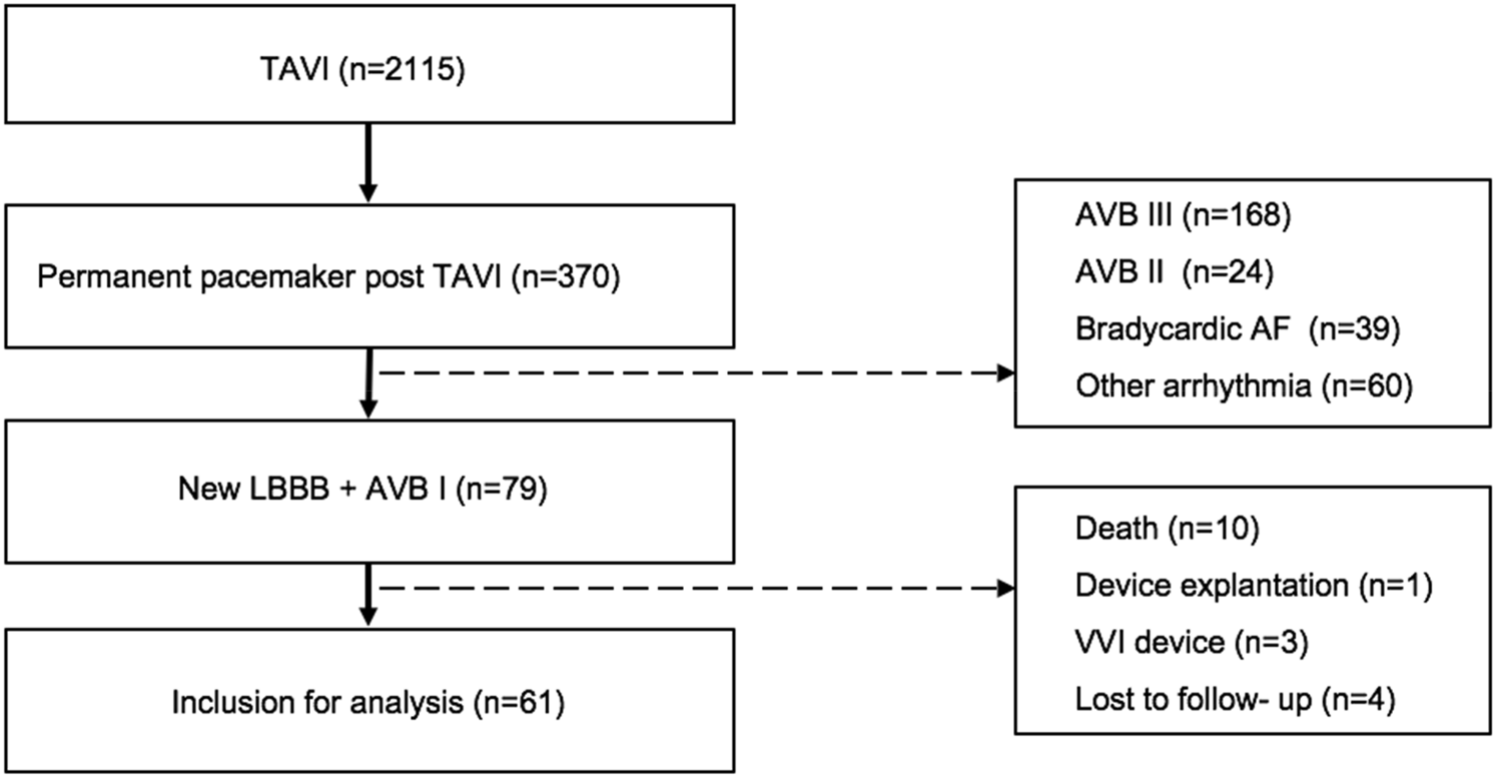
Flow chart of patients for analysis.

Between 2014 and 2018, 2115 patients received a transfemoral aortic valve replacement at the University Hospital of Munich. 370 patients required PPM implantation after the procedure. 79 of these patients underwent PPM implantation for new LBBB and new AVB I post TAVI. Complete follow up could be obtained in 61 patients. (Fig. 1) The patients had a mean age of 81 ± 7 years, 36.7% were female. Further patient characteristics are depicted in Table 1. 28 Patients (35.4%) had a known history of atrial fibrillation at implantation. At discharge, 57 patients (72.1%) were on betablocker medication.

**Table 1:** Baseline characteristics of patients with new onset LBBB and AVB I after TAVI.

Baseline characteristics	n = 79	
Age [years]	81.0 ± 7.3	
Male gender	50	63.3%
Hypertension	72	91.1%
Diabetes mellitus	22	27.9%
Chronic kidney failure	40	50.6%
Left ventricular ejection fraction [%]	55.2 ± 11.0	
Coronary artery disease	51	64.6%
Atrial fibrillation	28	35.4%
Incomplete right bundle branch block	20	25.3%
Betablocker	57	72.1%
Antiarrhythmic medication	4	5.0%

### Procedural characteristics

The valve prosthesis types used were *Sapien S3* (n = 44, 72.1%), *Core Valve* (n = 4, 6.6%), *Lotus* (n = 11, 18.0%) and *Acurate NEO* (n = 2, 3.3%). Prothesis type and size distribution were similar in both groups. The pacemaker devices used were *Biotronik Ecuro* (n = 5, 8.2%), *Biotronik Effecta* (n = 25, 41.0%), *Medtronic Ensura* (n = 16, 26.2%), *Medtronic Relia* (n = 1, 1.6%), *Medtronic Sensia* (n = 3, 4.9%), *Sorin Reply DR* (n = 8, 13.1%) *Sorin Koria* 100 (n = 1, 1.6%) and *Boston Advantio* (n = 2, 3.3%). Median time to PPMI after TAVI was 7 [3–9]days.

### Pacing rates and pacemaker settings

**Figure 2:**
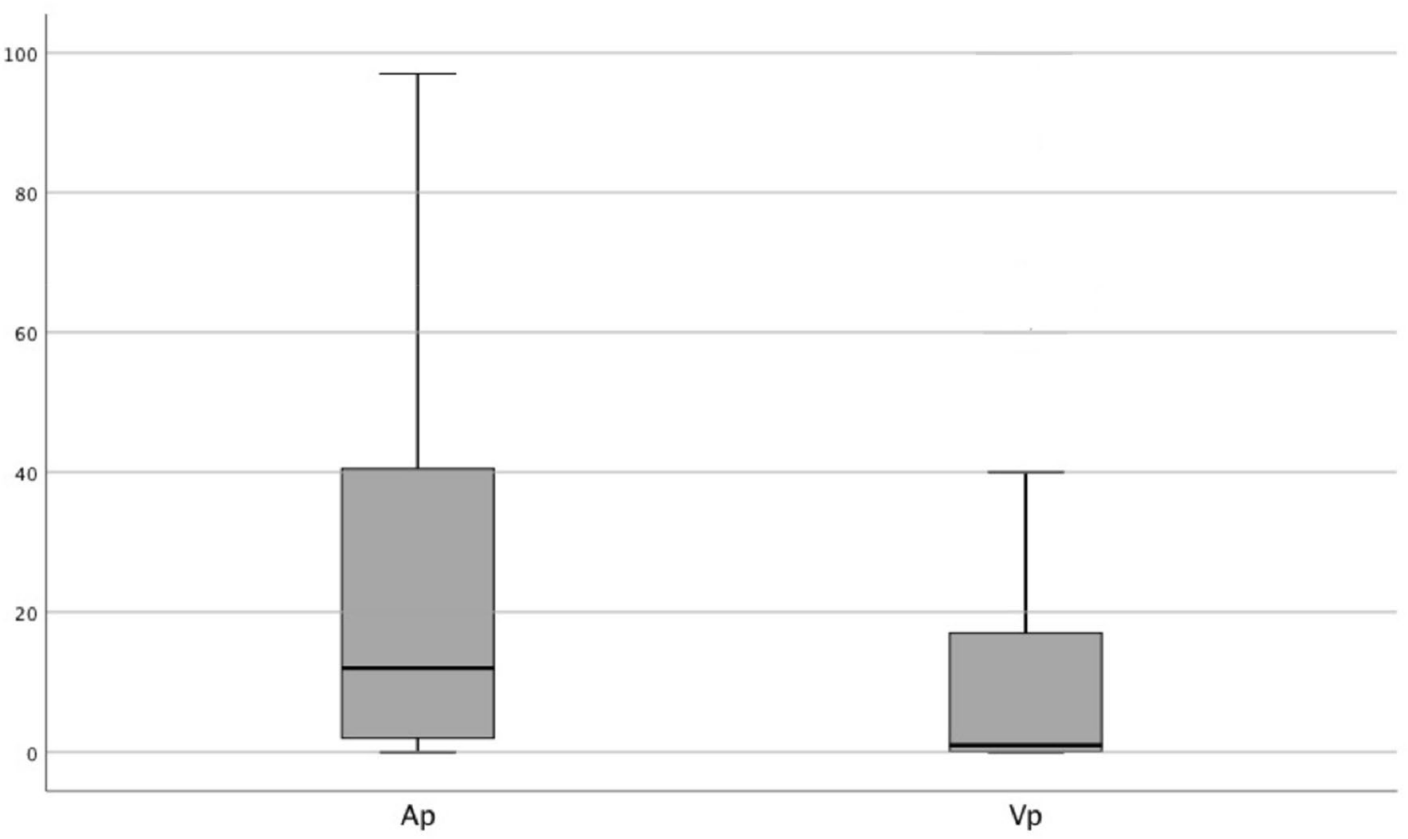
Overall atrial (a_p_) and ventricular pacing (v_p_) frequencies [%] at first outpatient pacemaker interrogation.

At the first pacemaker interrogation (median follow up at 6.23 [2.8–14.8] months), median overall Vp was 1.0% [0.1–17.8], Ap was 11.1% [1.0–40.8%] (Fig. 2). 59% of the patients (n = 36) had Vp ≥ 1%. The median Vp within the patient group with relevant ventricular pacing was 11.0% [1.0–64.0%].

Basic algorithms to reduce Vp were activated in most of the pacemaker settings (n = 60, 98%). ADI mode by Biotronik, managed ventricular pacing (MVP^®^) by Medtronic and SafeR^®^ by Sorin were considered potent Vp suppression algorithms. These were activated in 27 of the patients. Within the subgroup of patients with potent Vp suppression, 40.7% of patients (n = 11) required relevant ventricular pacing with a median Vp of 48% [4.0–91.0%].

### Predisposing factors for high ventricular pacing rates

Patients with irrelevant and relevant ventricular pacing showed similar age, comorbidities, valve size and medication at discharge (Table 2). *Edwards Sapien S3* was used significantly more often in patients who did not require relevant ventricular pacing at follow up *(88% vs. 61%, p* = *0.040).* The onset of new LBBB was early postprocedural (≤ 3d) in both groups. There was no significant difference in AV conduction times before and after TAVI between groups. However, patients requiring frequent ventricular pacing showed longer QRS duration (155 ms ± 17 ms vs. 144 ms ± 18 ms, p = 0.018) at time of pacemaker implantation. Numerically, more patients with relevant ventricular pacing had a history of atrial fibrillation, although this difference did not reach significance (38.9% vs. 16%, p = 0.086) (Table 3).

**Table 2:** Patient and procedural characteristics of both study groups.

	Vp < 1%(n = 25)		Vp ≥ 1% (n = 36)		p
Age [years]	78.4	± 8.4	81.3	± 6.4	0.165
Male gender	16	64.0%	24	66.7%	1.000
Hypertension	23	92%	32	89.0%	0.638
Diabetes mellitus	8	34.8%	11	30.6%	0.780
Chronic kidney disease	12	50.0%	17	47.2%	1.000
Coronary artery disease	17	68.0%	21	58.3%	0.592
Atrial fibrillation	4	16.0%	14	38.9%	0.086
**Aortic valve prosthesis type**					
Core Valve	0	0.0%	4	11.1%	0.137
*Sapien S3*	*22*	*88.0%*	*22*	*61.1%*	*0.040*
Lotus	2	8.0%	9	25.0%	0.106
Acurate NEO	1	4.0%	1	2.8%	1.000
Valve size [mm]	26.7	± 2.2	26.9	± 2.1	0.637
STS Score	5.5	± 4,7	3.7	± 1.8	0.113
Implantation depth [mm]	9.3	± 3.7	11.1	± 2,9	0.205

Significant values are in italic.

**Table 3:** Electrophysiological characteristics and pacemaker settings.

	Vp < 1%(n = 25)		Vp ≥ 1% (n = 36)		p
Incomplete RBBB	4	16.0%	5	13.9%	1.000
PQ post TAVI	246	235–292	254	238–284	0.857
Delta PQ (pre- and post TAVI)	59	26–90	53	31–67	0.849
*QRS post TAVI*	*144*	± *18.0*	*155*	± *17.4*	*0.018*
Delta QRS (pre- and post TAVI)	46	35–60	53	27–69	0.757
**Pacemaker mode**					
*Vp reduction algorithm (ADI, MVP®, Safe R®)*	*16*	*64%*	*11*	*30.6%*	*0.018*
AV-hysteresis	9	36.0%	23	63.8%	0.040

Significant values are in italic.

## Discussion

Several studies have demonstrated an increased risk for cardiac death in patients with new LBBB after TAVI. As a consequence, a relevant percentage of PPM implantations are for preventive reasons for LBBB and AVB I^8,10^. Taking into account that PPM implantation after TAVI poses an additional risk for implantation- related complications and has been associated with decreased left ventricular function^6,14^, the indication for PPM implantation should be carefully considered. In particular, there is still controversy whether patients with new LBBB with or without AVB I should receive a PPM. Under the prospect of a growing number of young patients receiving TAVI, the answer to this question is of increasing clinical relevance.

Previous data on pacemaker dependency after TAVI show persistent pacemaker dependency for patients with high degree AVB after TAVI. There are two earlier studies on ventricular pacing rates after TAVI that contained data on patients with new LBBB and new AVB I. In both investigations, patients with any indication for PPM implantation were included for analysis. The subgroups of patients with new LBBB and AVB I were comparatively small with 23 and 10 patients respectively. With Vp of 50% and 0% in the particular subgroup the two studies show conflicting results^12,13^. This study is the largest investigation to date with primary focus on pacing rates in patients with new LBBB and AVB I after TAVI, while addressing possible predisposing factors for high Vp in this collective.

Our results show that at follow up 6 months after implantation 59% of patients with new LBBB and AVB I require more than 1% ventricular pacing. Up to this day, there are only three further studies on pacemaker dependency following TAVI that also included patients with new LBBB and new AVB I. In all of these studies, this subgroup was underrepresented with only few patients with LBBB and AVB I enrolled and outcomes were variable^11–13^. The largest study so far enrolled a collective of 23 patients with LBBB and AVB I. Consistent with our study, the latter study found that 50% of patients require more than 1% Vp.

The cut off value of 1% ventricular pacing as a threshold for relevant pacemaker dependency is in line with previously published studies^11^. Whether the threshold of 1% can also be applied to a patient population that has received a PPM for preventive reasons, of course, remains an open question. We therefore also evaluated pacemaker settings to identify if ventricular pacing could have been avoided and analysed the subgroup of patients with potent Vp suppression algorithms. More than 40% of patients with potent Vp suppression algorithms had relevant ventricular pacing rates ≥ 1%. Median pacing frequency was high with 48%, thus supporting our findings that even with optimized pacemaker settings, a relevant proportion of patients requires ventricular pacing at follow-up. For the reasons mentioned above, however, this retrospective study is not able to clarify a pacemaker dependency in patients with new LBBB and AVB I with absolute certainty. The results give reason to assume that a relevant number of patients will benefit from PPM implantation if selected carefully.

By comparison, patients with and without relevant ventricular pacing showed significant difference in valve type and QRS duration after TAVI. There was a trend towards more frequent atrial fibrillation in patients with relevant ventricular pacing that was not significant.

The predominantly used prosthesis types in this study were Edwards Sapien, Core Valve, Lotus and Accurate Neo. Edwards Sapien was used significantly more often in patients with low ventricular pacing rates. In a recently published study by Chamandi et al., the use of Edwards Sapien was similarly associated with lower rates of PPM implantation at 30 days after TAVI^14^. In our cohort, Edwards Sapien valves were the only balloon- expandable valves used. Multiple previous studies could show that balloon expandable valves are associated with better clinical outcome und fewer pacemaker implantations^14–16^. Our results thus support earlier findings. Valve positioning and implantation technique were not taken into account for our analysis.

QRS-durations after TAVI were significantly longer in patients with relevant ventricular pacing. Median QRS duration after TAVI was 155 ms in patients with Vp ≥ 1% and 144 ms in patients with Vp ≤ 1% (p = 0.018). This is in line with earlier findings. A QRS-duration of more than 150 ms has been associated with higher risk of further conduction disturbances previously^17^. Proposals for management of new conduction disturbances after TAVI therefore recommend invasive electrophysiological testing, further ECG monitoring or PPM implantation for patients with QRS ≥ 150 ms and/or PQ ≥ 240 ms^18^.

In this study, patients with previous episodes of paroxysmal atrial fibrillation showed a mildly, but not significantly increased risk of pacemaker dependency. Patients with new LBBB and new AVB I after TAVI have been proven to show a high arrhythmic burden within the first year after the procedure. Both pre-existing and new onset atrial fibrillation have been reported to cause PPM implantation due to bradyarrhythmic events in up to one fifth of these patients^19^. Due to the retrospective design of this study, it was not possible to discern whether the increased pacing rates result from bradycardic episodes due to atrial fibrillation or whether the patients were more likely to develop further conduction disorders. Of course, the possibility of confounding due to anti- arrhythmic medication in this population needs to be considered. However, at hospital discharge, dosages of betablocker and antiarrhythmic medication were comparable between the two groups.

Similar to the overall cohort, patients with potent Vp suppression and relevant pacing rates had longer QRS durations after TAVI, [150 ms ± 14.8 ms vs 145 ms ± 21.4 ms], suffered more frequently from atrial fibrillation [36.4%(n = 4) vs 18.8%(n = 3)] and were less likely to have an Edwards Sapien prosthesis [63.6% (n = 7) vs 81.3% (n = 13)]. However, these trends did not reach significance.

Atrial pacing rates show high variability depending on pacemaker settings and medication and were therefore not a central subject of this study. It must be addressed that at a median of 11%, Ap was higher than could be expected in the collective. A subgroup of patients with new LBBB and new AVB I showed bradycardic episodes during the monitoring period. Although the additional sinus node disease would not have been an independent indication for PPMI before TAVI, it potentially resulted in higher Ap. However, after exclusion of these patients no changes in pacing rates and predisposing factors could be observed. (see supplementary Fig. 1 and Table 1).

## Limitations

This study has been designed as a retrospective investigation and as such is subject to possible limitations. Of course, this study is limited in its power to assess the need for PPM implantation. Further, prospective studies will be necessary to determine which patients with new LBBB and AVB I after TAVI benefit from PPM implantation. Due to comorbidities and prolonged rehabilitation a relevant amount of patients had delayed follow up visits. 18 patients did not complete follow-up at the study center. In addition, no data could be obtained on (1) medication at ambulatory presentation, (2) arrhythmic episodes and (3) clinical presentation. Moreover, although the study has been conducted at a large university hospital, the patient population is comparatively small. Predisposing factors for high ventricular pacing rates could therefore only been analysed in a descriptive way. Finally, both choice of implanted device and pacemaker settings were not standardized but were subject to the decision of the treating physician.

## Conclusion

This study poses the largest investigation on pacemaker dependency in patients with new LBBB and new AVB I after TAVI. At four months of follow-up, 59% of patients required ventricular pacing > 1%. Longer QRS duration of new complete LBBB, and implantation of a Non-Edwards-Sapiens-Valve were predisposing factors for relevant ventricular pacing. There was also a trend of more frequent atrial fibrillation in patients with relevant pacing rates.

## Supplementary Information

Supplementary Information.

## Data Availability

Department of Medicine I, University Hospital Munich, Marchioninistr. 15, DE-81377 Munich, Germany.

## Code availability

IBM® SPSS Statistics Version 25.0.
