# Supplementary material for: Permanent pacemaker dependency in patients with new left bundle branch block and new first degree atrioventricular block after transcatheter aortic valve implantation
Source: Sci Rep. 2021 Dec 21;11:24383. doi: 10.1038/s41598-021-03667-0 (PMC8692410; doi:10.1038/s41598-021-03667-0)
Supplement: Supplementary file 1 — Supplementary Information. [file 41598_2021_3667_MOESM1_ESM.pdf]

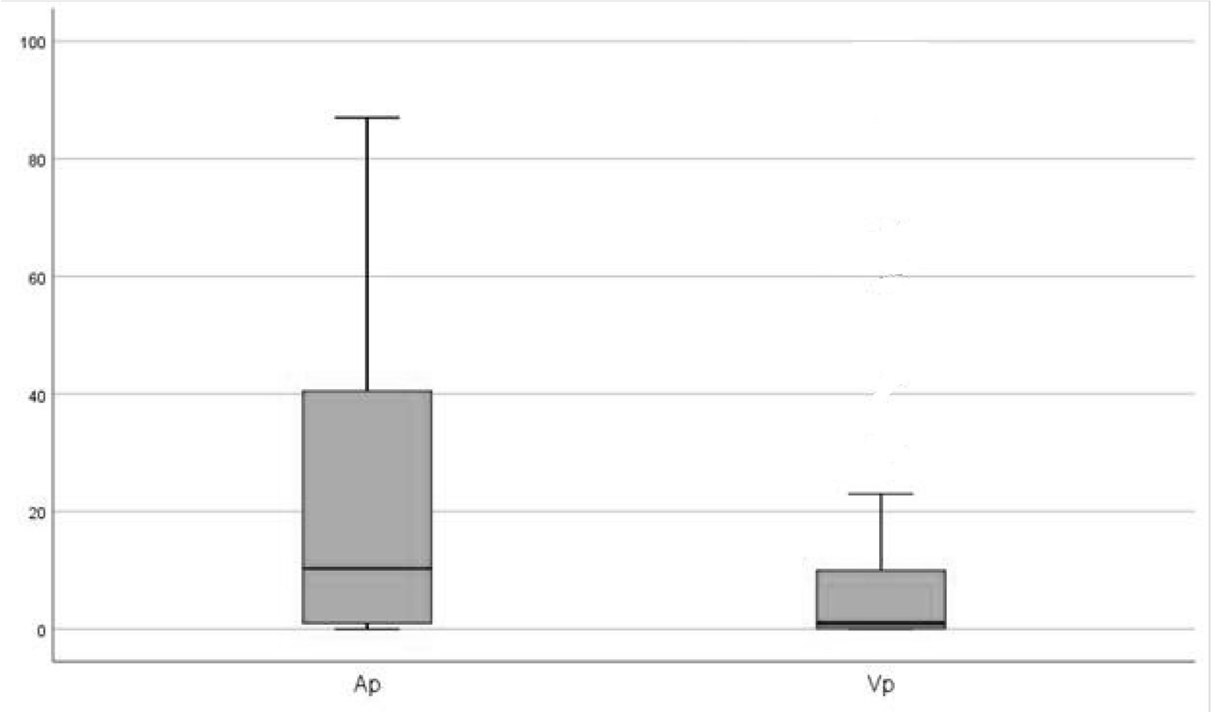

Supplementary Fig. 1: atrial (Ap) and ventricular (Vp) pacing rates after exclusion of patients with bradycardic episodes

	Vp <1%(n= 23)			Vp≥ 1% (n=31)			p
Age [years]	79.6	±7.0		82,9	±6.3		0.087
Male Gender	15	65.2 %		19	61.3%		0.768
Hypertension	21	91.3%		26	83.8%		0.596
Diabetes mellitus	6	26.1%		9	29%		0.811
Chronic kidney disease	11	47.8%		19	61.3%		0.574
Coronary Artery Disease	14	60.9%		20	64.5%		0.873
Atrial fibrillation	*3*	*13%*		*12*	*38.7%*		*0.064*
Aortic Valve Prosthesis Type							
Core Valve	0	0%		3	9.7%		0.253
Sapien S3	*20*	*87%*		*19*	*61.3%*		*0.037*

Lotus	2	8.7%		8	25.8%		0.161
Acurate NEO	1	4.3%		1	3.2%		1.000
Valve size [mm]	26,6	± 2,3		26,8	±2,3		0.812
STS Score	5.1	±4,7		4.6	±3,0		0.711
Incomplete RBBB	4	17.4%		6	19.4		0.854
PQ post TAVI	245	223-281		261	240-286		0.698
Delta PQ (pre- and post TAVI)	56	34-86		53	28-66		0.172
QRS post TAVI	*144*	*±19*		*156*	*±16*		*0.011*
Delta QRS (pre- and post TAVI)	47	37-73		52	24-72		0.779
Pacemaker mode							
Vp Reduction algorithm (ADI, MVP®, Safe R®)	*15*	*65.2%*		*9*	*29%*		*0.026*
AV- Hysteresis	8	34.7%		19	61.3%		0.098

Supplementary Tbl. 1 : Characteristics of patients with new onset LBBB and AVB I after TAVI, after exclusion of patients with bradycardic episodes
